# Arabinosylated Lipoarabinomannan Skews Th2 Phenotype towards Th1 during *Leishmania* Infection by Chromatin Modification: Involvement of MAPK Signaling

**DOI:** 10.1371/journal.pone.0024141

**Published:** 2011-09-14

**Authors:** Parna Bhattacharya, Gaurav Gupta, Saikat Majumder, Anupam Adhikari, Sayantan Banerjee, Kuntal Halder, Suchandra Bhattacharya Majumdar, Moumita Ghosh, Shubho Chaudhuri, Syamal Roy, Subrata Majumdar

**Affiliations:** 1 Division of Molecular Medicine, Bose Institute, Kolkata, India; 2 Division of Infectious Diseases and Immunology, Indian Institute of Chemical Biology, Council of Scientific and Industrial Research, Kolkata, India; French National Centre for Scientific Research - Université de Toulouse, France

## Abstract

The parasitic protozoan *Leishmania donovani* is the causative organism for visceral leishmaniasis (VL) which persists in the host macrophages by deactivating its signaling machinery resulting in a critical shift from proinflammatory (Th1) to an anti-inflammatory (Th2) response. The severity of this disease is mainly determined by the production of IL-12 and IL-10 which could be reversed by use of effective immunoprophylactics. In this study we have evaluated the potential of Arabinosylated Lipoarabinomannan (Ara-LAM), a cell wall glycolipid isolated from non pathogenic *Mycobacterium smegmatis*, in regulating the host effector response via effective regulation of mitogen-activated protein kinases (MAPK) signaling cascades in *Leishmania donovani* infected macrophages isolated from BALB/C mice. Ara-LAM, a Toll-like receptor 2 (TLR2) specific ligand, was found to activate p38 MAPK signaling along with subsequent abrogation of extracellular signal–regulated kinase (ERKs) signaling. The use of pharmacological inhibitors of p38MAPK and ERK signaling showed the importance of these signaling pathways in the regulation of IL-10 and IL-12 in Ara-LAM pretreated parasitized macrophages. Molecular characterization of this regulation of IL-10 and IL-12 was revealed by chromatin immunoprecipitation assay (CHIP) which showed that in Ara-LAM pretreated parasitized murine macrophages there was a significant induction of IL-12 by selective phosphorylation and acetylation of histone H3 residues at its promoter region. While, IL-10 production was attenuated by Ara-LAM pretreatment via abrogation of histone H3 phosphorylation and acetylation at its promoter region. This Ara-LAM mediated antagonistic regulations in the induction of IL-10 and IL-12 genes were further correlated to changes in the transcriptional regulators Signal transducer and activator of transcription 3 (STAT3) and Suppressor of cytokine signaling 3 (SOCS3). These results demonstrate the crucial role played by Ara-LAM in regulating the MAPK signaling pathway along with subsequent changes in host effector response during VL which might provide crucial clues in understanding the Ara-LAM mediated protection during *Leishmania* induced pathogenesis.

## Introduction

The parasitic protozoan *Leishmania donovani*, the causative agent of visceral leishmaniasis (VL), resides and multiplies in host macrophages [1]. Activated macrophages eliminate the intracellular parasite [2] by initiating host-protective anti-leishmanial responses. To counteract these anti-leishmanial responses parasites deploys different immune evasion strategies to survive within the macrophages [3]. *Leishmania*-induced macrophage dysfunctions have been correlated mainly with depletion of microbicidal molecules (nitric oxide (NO) and reactive oxygen species) [4], [5], and altered signaling events resulting in skewing the T helper cells (Th) to disease promoting Th2 subset that consequently suppress the host protective Th1 subset [6]. The outcome of infection in leishmaniasis is mainly determined by the Th1 versus Th2 effector response and the generation of IL-12 and IL-10 by the infected macrophages is important for this decision [7].

Several immunoprophylactics have been pursued to combat VL, with varying degrees of success. Recently Arabinosylated-lipoarabinomannan (Ara-LAM), an immunomodulator has emerged as an effective immunoprophylactic tool against VL. It confers protection during leishmanial pathogenesis via Toll-like receptor 2 (TLR2) signaling mediated nuclear factor-κ*B* (NF-κB) translocation and concomitant induction of the proinflammatory mediators [8].

In addition to NF-κB activation, TLR signaling can also activate mitogen-activated protein kinases (MAPK) signaling cascades which include extracellular signal–regulated kinase (ERKs), p38 MAPKs, and c-Jun NH2-terminal kinases (JNK) [9]. Most of the effector functions in response to extracellular cues are regulated by (MAPK) [10], [11]. The parasite-triggered reciprocal MAPK signaling via p38MAPK and ERK1/2 govern the counteracting immune response of the host cell resulting in differential expression of IL-12 and IL-10 in macrophages during *Leishmania* infection [12]. p38 MAPK activation results in histone modifications at the IL-12p40 promoter loci, making it more accessible for the recruitment of NF-kB leading to transcriptional induction of IL-12 [13]. In contrast, enhanced IL-10 transcription is associated with ERK1/2 activation leading to phosphorylation and acetylation of histone H3 at the IL-10 promoter loci which facilitates the binding of Signal transducer and activator of transcription 3 (STAT3) to the IL-10 promoter resulting in enhanced IL-10 transcription [14]. Moreover activated STAT3 attenuates the transcription of proinfllammatory mediators with the help of Suppressor of cytokine signaling 3 (SOCS3) inductions [15], [16], [ and 17]. Previous work from our laboratory has shown that Ara-LAM is involved in IL-12 induction and IL-10 attenuation during infection demonstrating the suitability of it as a potential candidate for immunotherapy to cure VL. But, how Ara-LAM treatment of parasitized macrophages leads to epigenetic modification at the locus of these two counteractive cytokine genes leading to their transcriptional regulation and the involvement of MAPK signaling in this regard is yet to be explored.

In the present study, we have found that Ara-LAM, a TLR-2 ligand confers protection against leishmanial pathogenesis via reciprocal regulation of MAPK signaling. This Ara-LAM mediated regulation of MAPK signaling resulted in antagonistic regulation of IL-12 and IL-10 in host macrophages. Detailed investigation at the molecular level showed that Ara-LAM could induce IL-12 by selective phosphorylation and acetylation of histone H3 residues at the IL-12p40 promoter region while attenuated IL-10 production by abrogating such histone H3 modification at IL-10 promoter in parasitized macrophages. This antagonistic regulation of effector response by Ara-LAM in the form of IL-10 and IL-12 was further linked to STAT3 and SOCS3 which were found to be crucial in regulating the host protective immune response in *leishmania* infected macrophages.

## Results

### 1. ERK and p38 MAP kinases differentially regulate Ara-LAM-mediated generation of macrophage effector molecules in *Leishmania donovani* infected macrophages

**Figure 1:**
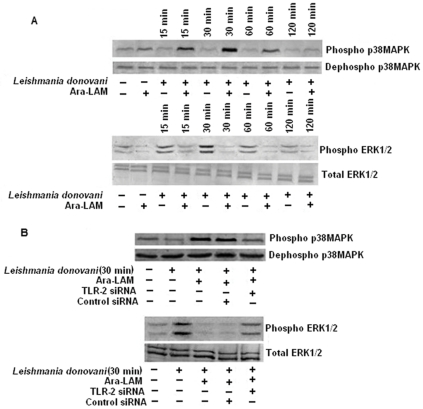
Ara-LAM mediated changes in MAPK signaling cascade in *Leishmania donovani*–infected macrophages. *Macrophages were pretreated with Ara-LAM for 3 hr or in some cases followed by Leishmania infection for 15, 30, 60, and 120 min. The cells were then lysed and subjected to Western blot with anti-pp38MAPK, p38MAPK and pERK1/2, ERK1/2, as described in Materials and Methods (A). In separate experimental sets, cells were transfected with control siRNA or TLR2-specific siRNA for 24 hr washed and then treated with Ara-LAM followed by L. donovani for 30 min. Western blot analysis was performed to analyze the expression of anti-pp38MAPK, p38MAPK and pERK1/2, ERK1/2 (B).*

**Figure 2:**
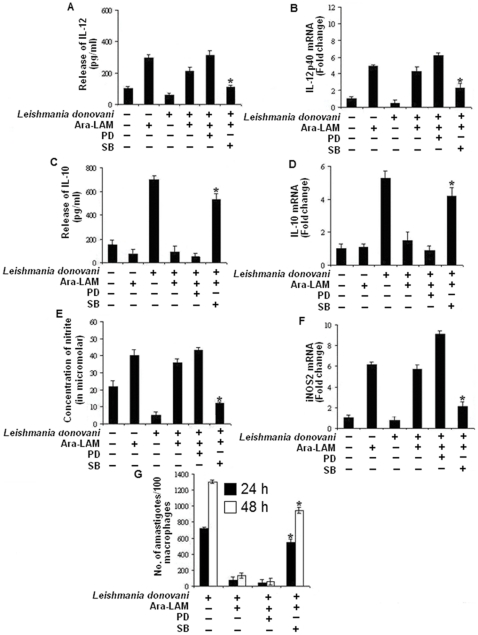
Ara-LAM mediated effector function depends on the reciprocal activation of p38MAPK and ERK–1/2. Peritoneal macrophages (2×10^6^cells/mL) were treated with SB203580 (SB) or PD098059 (PD) for 2 h, followed by Ara-LAM treatment for 3 hr. The cells were then infected with Leishmania parasite for 24 h and assayed for the levels of IL-12 (A) and IL-10 (C) in the culture supernatant by ELISA as described in Methods. ELISA data are expressed as means standard deviations of values from triplicate experiments that yielded similar observations. Macrophages cultured in a 24-well plate (1×10^6^ cells/mL) were pretreated and infected as described above and assayed for the levels of extracellular NO as described in the Materials and Methods (E). Asterisks indicate statistically significant induction of nitrite generation, compared with Ara-LAM–pretreated infected macrophages. *P<.001. From a separate set of cells (2×10^6^ cells/mL) RNA was isolated and levels of mRNA expression for IL-12p40 (B), IL-10 (D), inducible nitric oxide synthase 2 (iNOS2) (F) were determined by quantitative RT-PCR. Results are presented as changes (nfold) relative to uninfected control cells. The data represent the mean values± standard deviation of results from 3 independent experiments that all yielded similar results. In a separate experiment, the macrophages were cultured in coverglasses treated with SB or PD for 2 h, subsequently followed by 3 hr of Ara-LAM treatment and 4 h of Leishmania infection. After indicated time of incubation intracellular parasite number were assessed as described in methods. Pretreatment with SB significantly inhibited Ara-LAM –mediated parasite killing compared with levels in corresponding Ara-LAM pretreated infected controls (G). *P<.001 for SB.

Ara-LAM has been reported to confer protection against leishmanial pathogenesis via TLR2 signaling–mediated induction of the proinflammatory response [8]. However, it is unclear whether Ara-LAM can modulate the p38 and ERK1/2 MAPK signaling molecules which play differential role in the leishmanial pathogenesis [12]. We found that at an early time point, Ara-LAM stimulated phosphorylation of p38MAPK was much higher than infected macrophages; in contrast, ERK1/2 phosphorylation was abrogated in Ara-LAM treated parasitized macrophages compared to that in infected macrophages (figure **1A**). Interestingly, gene silencing of TLR-2 in infected macrophages reverses the Ara-LAM mediated regulation of MAPK family (figure **1B**). The MAPKs are key regulators of IL-10, IL-12 generation and NO production [18], [19]; leishmanial parasite leads to impaired effector response by suppressing p38MAPK induced IL-12, NO secretion while augmenting ERK-1/2 induced IL-10 production [12]. As Ara-LAM leads to significant protection during *leishmania* infection via a Th1 polarized anti-parasitic response [8], we further probed Ara-LAM induced leishmanicidal activity in the presence of p38MAPK and ERK inhibitors. Interestingly, preincubation of cells with PD098059 (an ERK inhibitor) followed by Ara-LAM treatment in parasitized macrophages caused slight increase in host protective IL-12 (figure **2A, 2B**) and NO generation (figure **2E, 2F**) along with concomitant decrease in IL-10 production (figure **2C, 2D**) both at the protein and mRNA level resulting in reduction in intracellular parasite load compared to Ara-LAM treated parasitized macrophages (figure **2G**). In contrast SB203580 (p38 MAPK inhibitor) treatment of infected macrophages almost completely abrogated Ara-LAM induced generation of IL-12 (figure **2A,2B**) and NO (figure **2E,2F**) whilst enhancing IL-10 production (figure **2C,2D**) both at the protein and mRNA level resulting in drastic increase in intracellular parasite load (figure **2G**), compared to Ara-LAM treated parasitized macrophages. In contrast, treatment of cells with the drugs (PD098059 and SB203580) after adding Ara-LAM and parasite resulted in similar levels of IL-12 p40, NO and IL-10 (both at the protein as well as the mRNA level) as compared to only Ara-LAM treated parasitized macrophages (data not shown). Interestingly, PD098059 and SB203580 itself had no effect on the levels of NO, IL-12p40 and IL-10 in Leishmania infected macrophages both at the protein as well as the mRNA level (figure **2**).

### 2. Ara-LAM stimulation of infected macrophages resulted in histone modifications and altered transcription factor binding at the IL-10 and IL-12 promoter region via reciprocal regulation of MAPK pathway

To get the mechanistic insight by which Ara-LAM reduces IL-10 and augmentes IL-12 synthesis during leishmanial pathogenesis, we analyzed various histone modifications at the IL-10 and IL-12 locus by Chromatin immunoprecipitation assay (CHIP).

**Figure 3:**
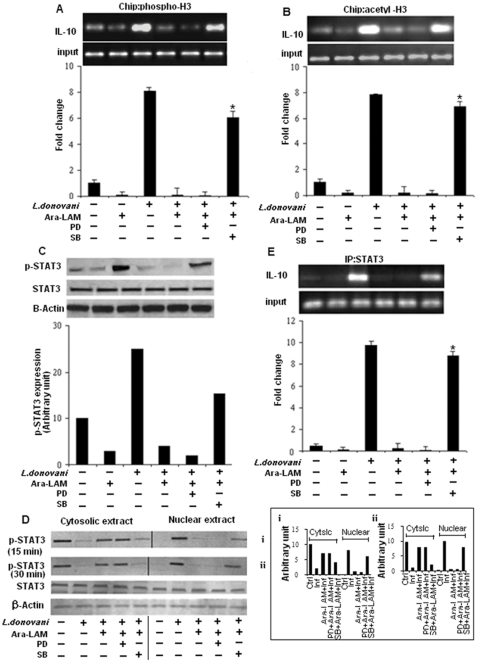
Histone H3 modifications at the IL-10 promoter in Ara-LAM treated infected macrophages. Murine macrophages (1×10^6^ cells/mL) were treated with SB or PD for 2 h, subsequently followed by Ara-LAM treatment for 3 hr and Leishmania infection for 45 min. After 45 min of incubation, ChIP assays were conducted as described in Materials and Methods. Immunoprecipitations were performed using Abs specific to phosphorylated H3 (IP phospho-H3) (A) or IP acetyl-H3 (B), and conventional RTPCR or quantitative real-time PCR was performed using primers specific to the IL-10 promoter. *P<.001 compared with Ara-LAM–pretreated infected macrophages. In a separate experiment, the macrophages were treated as described above followed by infection with *Leishmania parasite. After 24 hr of incubation, the cells were lysed and subjected to Western blot with anti-pSTAT3, and STAT3 as described in Materials and Methods (C). Peritoneal macrophages (3×10^6^ (cells/mL) were treated and infected by Leishmania for 15, 30 min as described in the legends of* *figure 1* . Cytosolic and nuclear protein extracts were prepared for Western blot analysis as described in material method to analyze the nuclear translocation of pSTAT3 and STAT3 (D). The blot shown is a representative of experiments performed in triplicate. Band intensities were analyzed by densitometry (i,ii). (inset). In a separate experiment murine macrophages (1×10^6^ cells/mL) were treated and infected with Leishmania parasite as described above. After 45 min of incubation, Immunoprecipitations were conducted using STAT3 (IP: STAT3) specific Abs. Conventional RT-PCR or quantitative real-time PCR was performed for amplifying the putative STAT3 binding sites of the IL-10 promoter. *P<.001 compared with Ara-LAM–pretreated infected macrophages.

High level of IL-10 production in leishmania-infected macrophages was found to be associated with increased histone H3 phosphorylation (figure **3A**) and acetylation (figure **3B**) at the IL-10 promoter which was abrogated in Ara-LAM pretreated parasitized macrophages. Interestingly, ERK inhibition prior to Ara-LAM treatment in parasitized macrophages slightly reduced both histone H3 phosphorylation (figure **3A**) and acetylation (figure **3B**) at IL-10 locus compared to only Ara-LAM treated parasitized macrophages. In contrast there was significant increase in histone H3 phosphorylation (figure **3A**) and acetylation (figure **3B**) at IL-10 locus in parasitized macrophages treated with both p38 inhibitor SB203580 and Ara-LAM, compared to only Ara-LAM pretreated parasitized macrophages. Because STAT3 is an obligate factor required for IL-10 gene transcription [20], we explored whether Ara-LAM could modulate the STAT3 expression during leishmanial infection. Ara-LAM pretreatment of host macrophages, abrogated parasite induced phosphorylation (figure **3C**) and nuclear translocation (figure **3D**) of STAT3 at early time points. Strikingly, inhibiting ERK resulted in decreased phosphorylation (figure **3C**) and subsequent nuclear translocation (figure **3D**) of STAT3 in Ara-LAM treated parasitized macrophages while p-38 inhibition reverses the phenomena and significantly increases the nuclear translocation of phosphorylated STAT3 compared to only Ara-LAM treated parasitized macrophages (figure **3C,3D**). CHIP assays were performed to examine the binding of STAT3 to the IL-10 promoter. Unstimulated control cells exhibited virtually no binding of STAT3 to the IL-10 promoter. Ara-LAM pretreatment significantly reduced the parasite induced binding of STAT3 to the IL-10 promoter (figure **3E**). Furthermore, inhibiting ERK prior to Ara-LAM treatment in infected macrophages, caused further decrease in STAT3 binding to the IL-10 promoter while inhibition of p38 in Ara-LAM treated macrophages resulted in significant increase in the binding of STAT3 to the IL-10 promoter when compared to only Ara-LAM treated infected sets (figure **3E**).

**Figure 4:**
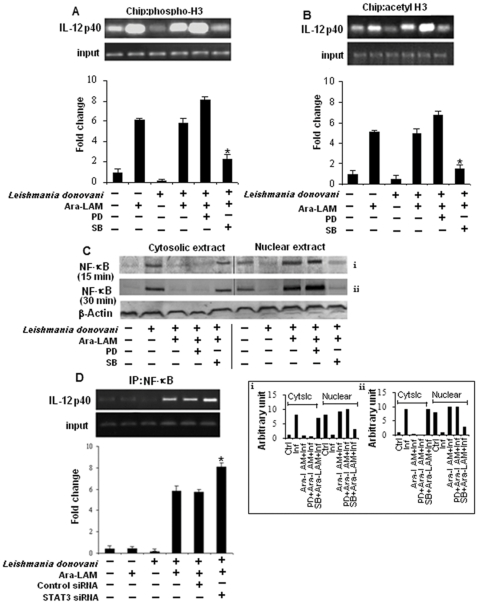
Hstone H3 modifications at the IL-12 promoter in Ara-LAM treated infected macrophages. *Murine macrophages (1×10^6^cells/mL) were treated and infected as described in the* *Figure 3* legend. After 45 min of incubation, ChIP assays were conducted as described in Materials and Methods. Immunoprecipitations were performed using Abs specific to phosphorylated H3 (IP phospho-H3) (A) or IP acetyl-H3 (B), and conventional RT-PCR or quantitative real-time PCR was performed using primers specific to the IL-12p40 promoter. *P<.001 compared with Ara-LAM–pretreated infected macrophages. In a separate experiment peritoneal macrophages were treated and infected by Leishmania for 15, 30 min as described in *Figure 3* legend. Cytosolic and nuclear protein extracts were analyzed for the nuclear translocation of NF-κB (C). The blot shown is a representative of experiments performed in triplicate. Band intensities were analyzed by densitometry (i,ii) (inset). Peritoneal macrophages (1×10^6^ cells/mL) were transfected with control siRNA or STAT3-specific siRNA, subsequently followed by Ara-LAM treatment (for 3 hr) and Leishmania infection for 45 min. Immunoprecipitations were conducted using NF-κB specific Abs. Conventional RT-PCR or quantitative real-time PCR was performed for amplifying the putative NF-κB binding sites of the IL-12p40 promoter. *P<.001 compared with Ara-LAM–pretreated infected macrophages.

Transcriptional induction of IL-12 gene requires histone modification of the promoter region, a mast for NF-kB recruitment, which plays a pivotal role in IL-12 transcription and is regulated by STAT3 [13], [15]. CHIP assay showed infected macrophages pretreated with Ara-LAM had higher levels of phosphorylated (figure **4A**) and acetylated (figure **4B**) H3 associated with IL-12p40 promoter relative to infected macrophages. Moreover, ERK inhibition in presence of Ara-LAM caused further increase in histone H3 phosphorylation (figure **4A**) and acetylation (figure **4B**) at IL-12p40 locus while p-38 inhibition significantly attenuated such Ara-LAM mediated histone modification at this locus in infected macrophages compared to only Ara-LAM treated parasitized macrophages (figure **4A,4B**).

Ara-LAM induced nuclear translocation of NF-kB was p38 dependent as p38 inhibition showed marked reduction in the nuclear translocation of NF-kB in infected macrophages (figure **4C**). Because, STAT3 plays a decisive role in the recruitment of NF-kB to the IL-12p40 promoter region [15], we investigated Ara-LAM mediated recruitment of NF-kB to the IL-12p40 promoter region under STAT3 silenced condition. There was significant enhanced recruitment of NF-kB to the IL-12p40 promoter in STAT3 knocked down macrophages, as compared to only Ara-LAM treated parasitized macrophages (figure **4D**), thereby pointing towards the critical role of STAT3 in down-regulating IL-12 gene expression in parasitized macrophages under Ara-LAM-treatment.

### 3. Ara-LAM induced attenuated SOCS3 expression is STAT3 dependent during VL

**Figure 5:**
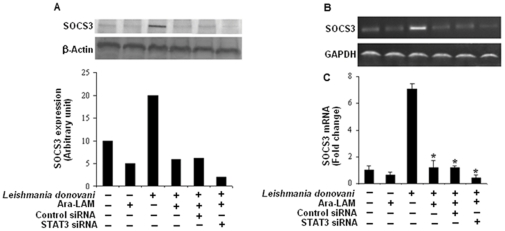
STAT3 silencing potentiate Ara-LAM mediated abrogation of SOCS3 expression in *Leishmania-donovani* infected macrophages. *Peritoneal macrophages (2×10^6^ cells/mL) were transfected with control siRNA or STAT3-specific siRNA, subsequently followed by Ara-LAM treatment (for 3 hr) and Leishmania infection for 24 hr. The cells were then lysed and subjected to Western blot with anti-SOCS3 antibody as described in Materials and Methods (A). In a separate experiment macrophages were transfected with either control-siRNA or* STAT3 specific siRNA, subsequently followed by Ara-LAM treatment for 3 hr and Leishmania infection for another 3 h. RNA was isolated and semi quantitative RT-PCR analyses for SOCS3 and GAPDH were done. Data represented here are from one of three independent experiments, all of which yielded similar results (B). Changes in expression of SOCS3 mRNA were also determined by quantitative real-time PCR. Results are presented as changes (n-fold) relative to uninfected control cells. The experiment was repeated 3 times, yielding similar results (C). *P<.001 compared with infected macrophages. The comparison of SOCS3 expression between Ara-LAM treated infected macrophages and STAT3 siRNA group did not show any statistical significance.

STAT3 activation plays a critical role in SOCS3 gene expression which in turn suppresses macrophage function [16]. As Ara-LAM treatment reduces the parasite induced STAT3 phosphorylation, we further studied its effect on SOCS3 expression, a downstream signaling intermediate of STAT3 pathway. Pretreatment of parasitized macrophages with Ara-LAM resulted in significant abrogation of the expression of SOCS3 both at the protein (figure **5A**) and m-RNA level (figure **5B, 5C**) compared to infected macrophages. To further examine whether Ara-LAM suppresses the expression of SOCS3 via the effective regulation of STAT3 signaling, we knock down STAT3 genes using STAT3 specific small interfering RNA (siRNA). STAT3 silencing of Ara-LAM treated parasitized macrophages resulted in higher reduction of SOCS3 expression as compared to only Ara-LAM treated parasitized macrophages which is not statistically significant. (figure **5A, 5B, and 5C**). These findings clearly suggest that Ara-LAM negatively regulates the STAT3 signaling leading to marked reduction in SOCS3 expression.

### 4. SOCS3 silencing significantly enhances host protective proinflammatory response and antigen presentation in Ara-LAM treated infected macrophages

**Figure 6:**
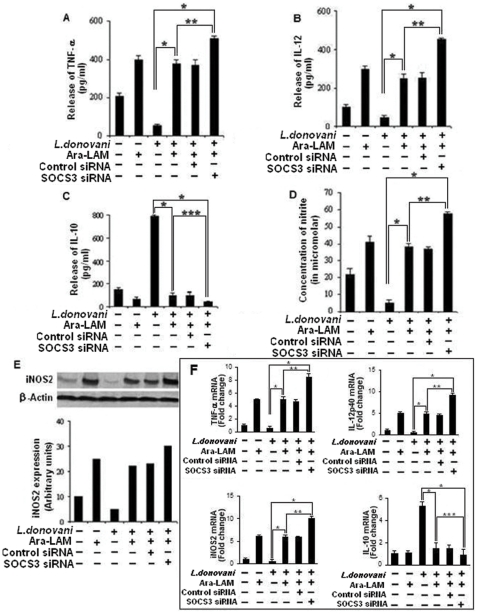
SOCS3 silencing significantly enhances host protective proinflammatory response generation by Ara-LAM in infected macrophages. Peritoneal macrophages (2×10^6^ cells/mL) were transfected with control siRNA or SOCS3-specific siRNA followed by Ara-LAM treatment (for 3 hr) and Leishmania infection for 24 h and assayed for the levels of TNF- α (A), IL-12 (B), and IL-10 (C) in the culture supernatant by ELISA, as described in Methods. ELISA data are expressed as means standard deviations of values from triplicate experiments that yielded similar observations. *P<.001 compared with infected macrophages, **P<.005 compared with Ara-LAM–pretreated infected macrophages. ***<.01 compared to Ara-LAM–pretreated infected macrophages. In a separate experiment macrophages were cultured and treated as described above and assayed for the levels of extracellular NO as described in the Materials and Methods (D). *P<.001 compared with infected macrophages, **P<.005 compared with Ara-LAM–pretreated infected macrophages. In a separate set macrophages were transfected and treated with Ara-LAM as described above followed by Leishmania infection for 24 hr. Western blot analysis was performed to analyze the expression of inducible nitric oxide synthase. The blot shown is a representative of experiments performed in triplicate. Band intensities were analyzed by densitometry (E). Peritoneal macrophages were cultured and treated with Ara-LAM as described above followed by Leishmania infection for 3 hr. Changes in mRNA expression of IL-12p40, TNF- α, IL-10, NO were determined by real-time PCR analysis Results are presented as changes (n-fold) relative to uninfected control cells. The experiment was repeated 3 times, yielding similar results; data are expressed as means ± standard deviations. (F). *P<.001 compared with infected macrophages, **P<.005 compared with Ara-LAM–pretreated infected macrophages.

Previous study with Ara-LAM showed that it could confer protection against leishmanial pathogenesis via induction of the proinflammatory response [8]. As SOCS3 has been speculated to participate in inhibition of macrophage activation [17], we tested whether SOCS3 interferes with Ara-LAM -stimulated signal transduction events in *Leishmania donovani* infected macrophages. We observed that SOCS3 silencing could markedly increase the Ara-LAM induced production of proinflammatory mediators such as IL-12 (figure **6B, 6F**), TNF-α, (figure **6A, 6F**) NO (figure **6D, 6E, 6F**) along with the downregulation of immunosupressive cytokine IL-10 (figure **6C, 6F**) in parasitized macrophages both at the protein and mRNA level. These results suggest that Ara-LAM leads to the production of host protective proinflammatory response by supressing SOCS3 functioning.

**Figure 7:**
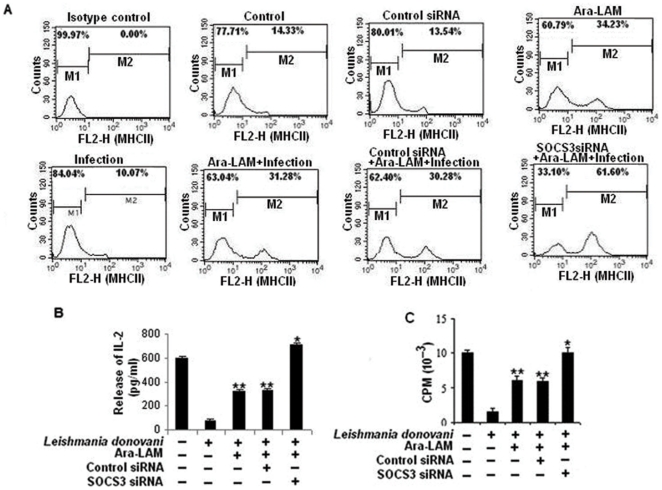
Ara-LAM induced MHC-II expression, antigen presentation ability of infected macrophages increased under SOCS3 silenced condition. *Peritoneal macrophages (2×10^6^ cells/mL) were transfected with control siRNA or SOCS3-specific siRNA, subsequently followed by Ara-LAM treatment (for 3 hr) and Leishmania infection for 4 hr as mentioned above. After 24 hr of incubation treated macrophages were analyzed by flow cytometry for MHCII (FL2-H) expression as described in material method (A). Data are from 1 of 3 experiments conducted in the same way with similar results. In a separate set, normal and treated macrohages either uninfected or infected were pulsed with λR_12–26_, and then were incubated with T-cell hybridoma 9H 3.5. The culture supernatants were analysed for the presence of IL-2 by ELISA as described in the methods (B). Incorporation of 3H-Thymidine in the IL-2 dependent cell line HT-2 was assessed in presence of the cultured supernatant. Results are expressed as mean* ± SD of 5 replicate experiments (C). *P<.001, **P<.005 compared with infected macrophages.

SOCS3 has also been reported to down regulate major histocompatibility -II (MHC-II) expression resulting in decreased antigen presentation by macrophages [21], a hallmark of leishmanial pathogenesis [22]. Fascinatingly, Ara-LAM treatment prominently restores MHC-II expression in *L. donovani*–infected macrophages (figure **7A**). We also investigated the role of Ara-LAM on the antigen presentation capability of infected macrophages by measuring their ability to activate λ peptide stimulated T cell hybridoma 9H3.5 cells in terms of IL-2 production which is important for T effector cell functioning. In case of infected macrophage, there was a significant impairment of antigen presentation compared with uninfected macrophages (figure **7C**) along with lower IL-2 secretion from T cell hybridoma 9H3.5 when co-cultured with infected macrophages (figure **7B**). Interestingly the release of IL-2 from 9H3.5 cells and antigen presentation ability could be effectively increased under Ara-LAM pretreatment in infected macrophages. (figure **7B,7C**). Moreover, parasitized macrophages in which SOCS3 was knocked down prior to Ara-LAM treatment resulted in enhanced MHC-II expression (figure **7A**) as reflected by greatly increased antigen presentation (figure **7C**) and higher IL-2 production from 9H3.5 cells (figure **7B**) demonstrating that SOCS3 interferes with antigen presentation and decreased SOCS3 was further potentiating Ara-LAM mediated antigen presentation ability of infected macrophages.

## Discussion

*Leishmania*-induced deactivation of macrophage functions are due to defects in the signaling pathways, which facilitates the parasite to infect and propagate within the cell [23]. Recently the capacity of *Leishmania* to suppress macrophage function has been linked to alterations in MAPK signaling cascades [12], [24], [25]. To revert this parasite induced changes in MAPK signaling, novel immunomodulators are being evaluated for their functionality in VL. Ara-LAM, a potential immunomodulator strongly initiates the host protective immune response during VL via TLR-2 mediated proinflammatory responses [8]. But its role in regulating the MAPK signaling pathway has not been explored till date.

Recently it has been demonstrated that the differential activation of the MAPKs regulates cytokine production. CD40, a costimulatory molecule with host-protective anti-leishmanial function, could induce both IL-10 and IL-12 by differential activation of p38 MAPK and ERK-1/2, respectively. This reciprocal regulation of p38 MAPK and ERK1/2 has been shown to be responsible for the protection *versus* susceptibility to infection [12], [24].

Ara-LAM pretreatment restored the impaired p38MAPK phosphorylation in parasitized macrophages and abrogated the ERK1/2 phosphorylation via TLR-2 mediated downstream signaling (figure **1**) thus shifting the macrophageal microenvironment from anti-inflammatory (IL-10) towards proinflammatory (IL-12) host protective immune response (figure **2**). Our finding is consistent with other studies which have also highlighted the involvement of this novel molecule, Ara-LAM, in inducing proinflammatory responses (IL-8, TNF-α) in macrophages via interacting with TLR-2 [26], [27]. Additionally, Ara-LAM has been demonstrated as a major component responsible for the pro-inflammatory activity of the whole bacteria (*M. smegmatis and M. fortuitum*) [28].

Having identified the reciprocal alteration in Ara-LAM mediated production of IL-12 and IL-10 during VL, we studied whether Ara-LAM could modulate the chromatin (specifically histone H3) at the locus of these two counteractive cytokine genes since covalent modification to histones of chromatin can lead to differential gene expression. Our study revealed that Ara-LAM pretreatment leads to IL-12 induction via histone H3 phosphorylation (figure **4A**) and acetylation (figure **4B**) of chromatin at IL-12 locus along with IL-10 attenuation by abrogating the parasite induced histone H3 phosphorylation (figure **3A**) and acetylation (figure **3B**) at IL-10 locus during leishmanial pathogenesis. Thus Ara-LAM mediated gene-specific chromatin modifications are found to be associated with transient silencing of IL-10 genes and priming of the IL-12 gene.

To further ascertain the role of MAPKs in the regulation of counteracting cytokines IL-10 and IL-12, we investigated Ara-LAM mediated transcriptional alteration of these cytokine genes in the presence of their respective pharmacological inhibitors. Our results implicated that the inhibition of p38 MAPK pathway in Ara-LAM treated parasitized macrophages abrogates chromatin remodeling and nuclear translocation of NF-kB thereby attenuating IL-12 transcription (figure 4A, 4B, 4C) and simultaneously reverses Ara-LAM mediated attenuated IL-10 production in parasitized macrophages via increased histone H3 phosphorylation, acetylation and enhanced STAT3 recruitment to the IL-10 promoter leading to the induction of IL-10 transcription (figure 3A,3B,3D,3E). In contrast ERK inhibition of Ara-LAM treated parasitized macrophages leads to further increase in IL-12 transcription (figure 4A, 4B, 4C) along with significant reduction of the IL-10 (figure 3A,3B,3D,3E) compared to Ara-LAM treatment only. Therefore, this study suggested a crucial role of MAPK in Ara-LAM mediated modulation of IL-12 and IL-10 gene transcription during leishmanial pathogenesis.

The signaling pathways which direct IL-10-mediated inhibition of IL-12 production at the level of transcription is well documented [12]. Recent observations have suggested that absence of STAT3 resulted in enhanced lipopolysacharide induced IL-12p40 gene expression in IL-10^_^_/_^_^ mice due to enhanced NF-κB recruitment to the IL-12p40 promoter [15]. Our observation also indicated that STAT3 silencing significantly enhances Ara-LAM mediated recruitment of NF-κB to the IL-12 p40 promoter (figure **4D**) indicating that STAT3 negatively regulates Ara-LAM mediated transcription of IL-12 gene during leishmanial pathogenesis.

Moreover, STAT3 activation leads to the induction of SOCS3, which plays a decisive role in inactivation of macrophage function [16]. SOCS3 is critical for the survival strategy of mycobacteria [29], leishmania [30], listeria [31] and RNA viruses [32] in the host macrophages. Moreover silencing of the SOCS3 gene resulted in a marked reversal of the immunosuppressive effect of IL-10 on many inflammatory mediators including cytokines, chemokines, and IFN-inducible, apoptotic and TNF ligand/receptor genes [33].

Ara-LAM treatment lead to significant reduction in SOCS3 expression by inactivation of STAT3 (figure **5A, 5B, 5C**). These changes resulted in significant induction of host protective proinflammatory responses (figure **6**), MHC-II expression, and antigen presentation capability of infected macrophages (figure **7A, 7C**) along with enhanced T cell effector functioning as evident from higher IL-2 production from co-cultured T cells (figure **7B**). Further confirmation about the role of SOCS3 in the generation of host protective immune responses in Ara-LAM treated infected sets was done by SOCS3 silencing which was suggestive of SOCS3 hindrance in Ara-LAM mediated induction of proinflammatory response (figure **6** **,** **7A, 7B, 7C**).

In summary, our current findings provide a detailed molecular mechanistic insight of Ara-LAM mediated IL-12 and IL-10 production in *Leishmania donovani* infected macrophages where MAPK signaling via differential regulation of p38 and ERK plays an important role in generating host protective immune response during leishmanial pathogenesis.

## Materials and Methods

### Reagents and Chemicals

RPMI-1640 medium, M-199 medium (M199), penicillin and streptomycin, SB203580 (p38MAPK inhibitor), PD098059 (ERK inhibitor) and TRI Reagent were from Sigma (St Louis, MO, USA). Fetal calf serum (FCS) was obtained from Gibco BRL (Grand Island, NY, USA) and ELISA Assay Kit (Quantikine M) for tumour necrosis factor (TNF)-α, IL-12, IL-10 were from R&D Systems (Minneapolis, MN, USA). dNTPs, RevertAidTM M-MuLV Reverse Transcriptase, oligo dT, RNase inhibitor and other chemicals required for cDNA synthesis were from Fermentas (USA). Anti-phospho-H3 and acetyl-H3 Abs were purchased from Abcam and chromatin immunoprecipitation (ChIP) assay kits were purchased from Millipore. SOCS3, STAT3 siRNA were procured from Santa Cruze biotech.

### T-cell hybridoma and lambda repressor peptide

9H3.5 (I-A^d^ restricted T-cell hybridoma) specific for lambda repressor N-terminal sequence 12–26 [LEDARRLKAIYEKKK, defined as λR _12–26_]. T-cell hybridomas and peptides were kind gifts of Professor M. L. Gefter, Massachusetts Institute of Technology, Cambridge, MA, USA. The IL-2 dependent cell line HT-2 (mouse T helper cell line) was obtained from American Type Culture Collection. All cells were maintained in RPMI 1640 medium supplemented with 10% fetal calf serum (FCS) and 2-ME (5×10^−5^ M) at 5% CO2 in humidified atmosphere.

### Animals and parasites

BALB/c mice were purchased from the National Centre for Laboratory Animal Sciences, India. For each experiment 8–10 mice (4–6 weeks old) were used, regardless of sex. *L. donovani* organisms (strain MHOM/IN/1983/AG-83) were maintained in Medium 199 (Sigma) plus 10% fetal calf serum (Gibco). Amastigotes were prepared as described elsewhere [34]. Stationary-phase promastigotes obtained by suitable transformation were used for experiments. All experimental protocols were given prior approval by the institutional animal ethics committee.

### Isolation and purification of Ara-LAM

Ara-LAM was isolated as described elsewhere [35] Lipopolysaccharide contamination was checked by the Limulus test and was <25 ng/mg in Ara-LAM. The noncytotoxic dose of Ara-LAM was 3 µg/mL [36].

### Peritoneal macrophage preparation

Macrophages were isolated by peritoneal lavage with ice-cold phosphate-buffered saline at 48 h after intraperitoneal injection of 1.0 mL of sterile 4% thioglycolate broth (Difco). Cells were cultured as described elsewhere [37].The adherent cell population was cultured for 48 h prior to any treatment, to achieve the resting state.

### Uptake and intracellular multiplication

For assessing the activity of Ara-LAM against the amastigote stage of parasite, peritoneal macrophages cultured on glass cover slips were pretreated with Ara-LAM (3 µg/ml) for 3 h, followed by infection with *L.donovani* promastigotes at a ratio of 1∶10 for the indicated time periods; macrophages were then fixed and stained as described elsewhere [38] for calculation of the number of intracellular parasites. When indicated, the uptake and multiplication of *L.donovani* was studied in the presence and absence of the p38MAPK inhibitor SB203580 (10 µg/mL), or ERK inhibitor PD098059 (100 µmol/L; Sigma) [12].

### Cytokine enzyme-linked immunosorbent assay (ELISA)

The conditioned medium of macrophage culture was assayed for mouse cytokines and chemokines with use of the sandwich ELISA kit (Quantikine M; R&D Systems). The assay was performed according to the manufacturer's instructions.

### Preparation of cell lysate and immunoblot analysis

Cell lysates were prepared as described elsewhere [39]. Equal amounts of protein (50 µg) were subjected to 10% sodium dodecyl sulfate polyacrylamide gel electrophoresis, and were subsequently transferred to a nitrocellulose membrane. The membrane was blocked overnight with 3% bovine serum albumin in Tris-saline buffer (pH, 7.5), and immunoblotting was performed to detect Inducible Nitric Oxide Synthase 2 (iNOS2) and phosphorylated or dephosphorylated forms of p38MAPK, ERK1/2, STAT3 and β-Actin as described elsewhere [40].

### Nitrite generation

Nitrite level in culture was measured using the Nitric Oxide Colorimetric Assay kit (Boehringer Mannheim Biochemicals) [41]. Cell-free supernatants were collected from different experimental sets at different time points of infection, and nitrite levels were estimated in accordance with the manufacturer's instructions. Data were expressed in micromoles of nitrite.

### Antigen presentation assay

Antigen presenting ability of infected macrophages was studied by their ability to present λR_12–26_ to T-cell hybridoma (9H 3.5). The antigen presenting cells (APC) were incubated for 24 h with specific peptide and T-cell hybridoma in complete RPMI medium in a 37°C incubator. The culture supernatants were analysed for the presence of IL-2 by growing an IL-2 dependent cell line HT-2 in the supernatants. HT-2 (10^4^ cells per well) was incubated with a 50% concentration of culture supernatant for 48 h. The cells were then pulsed with 1 µCi of ^3^H-Thymidine [6.7 Ci/mmole, New England Nuclear, Boston, NE, USA)] for the last 18 h [42]. The incorporation of radioactive thymidine was then assessed by a scintillation counter (Packard).

### Flow cytometry

BALB/c-derived macrophages were stained with phycoerythrin (PE) labeled anti-MHCII antibodies. The cells were analyzed by a FACSVantage™ flow cytometer (Becton Dickinson).

### Preparation of nuclear and cytoplasmic extracts

The nuclear extracts were prepared from normal and infected macrophages as described elsewhere [43]. Briefly, sedimented cells were resuspended in hypotonic buffer (10 mM HEPES (pH 7.9), 1.5 mM MgCl2, 10 mM KCl, 0.2 mM PMSF, and 0.5 mM DTT) and allowed to swell on ice for 10 min. Cells were homogenized in a homogenizer. The nuclei were separated by spinning at 3300×*g* for 5 min at 4°C. The supernatant was used as the cytoplasmic extract. The nuclear pellet was extracted in nuclear extraction buffer (20 mM HEPES (pH 7.9), 0.4 M NaCl, 1.5 mM MgCl2, 0.2 mM EDTA, 25% glycerol, 0.5 mM PMSF, and 0.5 mM DTT) for 30 min on ice and centrifuged at 12,000×*g* for 30 min. The supernatant was used as nuclear extract.

### CHIP assay

CHIP assays were conducted using the CHIP Assay kit following the manufacturers 

Protocol (Millipore). Briefly, 1×10^6^ peritoneal macrophages were plated overnight in six-well plates. Cells were stimulated as described in figures, and then fixed for 10 min at 37°C in 1% paraformaldehyde. Cells were washed on ice with ice-cold HBSS containing 1 mM PMSF, harvested and then lysed in SDS lysis buffer. DNA was sheared by ultrasonication using a High Intensity Ultrasonic Processor (hielscher) for 3×10 s pulses at 20% amplitude. Lysates were cleared by centrifugation and diluted in ChIP dilution buffer. Lysates were precleared using salmon sperm DNA/protein A-agarose and a sample of “input DNA” was collected at this point. Protein-DNA complexes were immunoprecipitated with 5 µg of Ab overnight at 4°C. Ab-protein-DNA complexes were then captured using salmon sperm DNA/protein A-agarose for 1 h at 4°C. After washing beads with low and high salt, LiCl, and TE buffers, the protein/DNA complexes were eluted using 1% SDS, 0.1 M NaHCO3 buffer and disrupted by heating at 65°C for 4 h. DNA was then extracted using phenol/chloroform extraction and ethanol precipitation. PCR was conducted using promoter specific primers: IL-10 promoter (STAT3 binding region): sense 5′-TCATGCTGGGATCTGAGCTTCT-3′, antisense 5′-CGGAAGTCACCTTAGCACTCAGT-3′ (94°C, 15 s; 56°C, 30 s; 72°C, 1 min, 35cycles); IL-12p40 promoter (NF-κB binding site): sense 5′-AGTATCTC TGCCTCCTTCCTT-3′, antisense 5′-GCAACACTGAAAACTAGTGTC-3′ (initial denaturation at 95°C for 3 min; amplification cycles at 95°C for 30 s, 58°C for 1 min, and 72°C for 1 min; and a final extension at 72°C for 10 min, 40cycles). PCR amplified product was subsequently size fractioned on 2% agarose gel, stained with ethidium bromide and visualized under UV-light. For relative quantitation of promoter levels, real-time PCR was also performed. For real-time PCR samples were normalized to input DNA controls.

### Isolation of RNA and real-time polymerase chain reaction

Total RNA extracted from macrophages (TRI reagent; Sigma) according to the standard protocol [44], [45] was reverse transcribed using Revert Aid M-MuLV reverse transcriptase (Fermentas). Real-time polymerase chain reaction (PCR) was performed using SYBR Green mix and the ABI 7500 real-time PCR system (Applied Biosystems). Glyceraldehyde- 3-phosphate dehydrogenase (GAPDH) was used as a reference. Sequences of the PCR primers are listed in Table 1. The reaction conditions consisted of an initial activation step (5 min at 95°C) and cycling step (denaturation for 30 s at 94°C, annealing for 30 s at 58°C, and extension for 1 min at 72°C for 40 cycles), after which melt curve analysis was performed. Detection of the dequenched probe, calculation of threshold cycles, and further analysis of these data were done using Sequence Detector software (version 1.4; Applied Biosystems). Relative changes in iNOS2 and cytokine messenger RNA (mRNA) expression were compared with unstimulated control, normalized to GAPDH, and quantified by the 2^ΔΔCt^ method.

**Table 1:** Sequences of the PCR primers.

Gene	Sequence of Primers
iNOS2	Forward 5′-CCCTTCCGAAGTTTCTGGCAGCAGC-3′Reverse 5′-GGCTGTCAGAGCCTCG TGGCTTTGG-3′
IL-10	Forward 5′-CGGGAAGACAATAACTG-3′Reverse 5′-CATTTCCGATAAGGCTTGG-3′
IL-12p40	Forward 5′-CAACATCAAGAGCAGTAGCAG-3′Reverse 5′TACTCCCAGCTGACCTCCAC-3′
TNF-α	Forward 5′-GGCAGGTCTACTTTGGAGTCATTGC-3′Reverse 5′-ACATTCGAGGCTCCAGTGAATTCGG-3′
SOCS-3	Forward 5′-GCGGGCACCTTTCTTATCC-3′Reverse 5′-TCCCCGACTGGGTCTTGAC-3′
GAPDH	Forward 5′-CAAGGCTGTGGGCAAGGTCA-3′Reverse 5′-AGGTGGAAGAGTGGGAGTTGCTG-3′

### Isolation of RNA and RT-PCR

RNA was isolated according to the standard protocol [44], [45]. Briefly, total RNA extracted from macrophages (TRI reagent; Sigma) according to the standard protocol [44], [45] was reverse transcribed using Revert Aid M-MuLV reverse transcriptase (Fermentas). The cDNA encoding the SOCS3, GAPDH gene was amplified using specific primers as listed in Table 1. PCR amplification was conducted in a reaction volume of 50 µl using a Perkin Elmer Gen Amp PCR system 2400 and 0.5 unit of Taq polymerase set for 35 cycles (denaturation: at 94°C for 30 s; annealing: at 58°C for 30 s; extension: at 72°C for 30 s) PCR amplified product was subsequently size fractioned on 2% agarose gel, stained with ethidium bromide and visualizedunder UV-light.

### Densitometry analysis

Immunoblots were analyzed using a model GS-700 Imaging Densitometer and Molecular Analyst (version 1.5; Bio-Rad Laboratories).

### Statistical analysis

The in vitro cultures were performed in triplicate. The data, represented as mean values ±standard deviations, are from 1 experiment that was performed at least 3 times. Student's t test was employed to assess the significance of the differences between the mean values of control and experimental groups. A P value of.05 was considered to be significant, and a P value <.001 was considered to be highly significant.

### Ethics Statement

This study was carried out in strict accordance with the recommendations in the Guide for the Care and Use of Laboratory Animals of the National Institutes of Health. All experimental animal protocols received prior approval from the Institutional Animal Ethical Committee (Bose Institute, Registration Number: 95/99/CPCSEA).
